# Advantages of Side-lying Position. a Comparative Study of Positioning During Bottle-feeding in Preterm Infants (≤34 Weeks GA)

**DOI:** 10.34763/jmotherandchild.20212504.d-22-00008

**Published:** 2022-06-09

**Authors:** Anna Raczyńska, Ewa Gulczyńska, Tomasz Talar

**Affiliations:** 1 Department of Neonatology, Pathology and Neonatal Intensive Care Unit, Polish Mother’s Memorial Hospital – Research Institute, Łódź, Poland

**Keywords:** premature, oral feeding, positioning, side-lying position (SLP)

## Abstract

**Background:**

The quality and safety of bottle-feeding in premature infants can be improved by optimal positioning. This study analysed the advantages of side-lying position (SLP) and semielevated position (SEP) during bottle-feeding in premature infants.

**Material and methods:**

A total of 42 neonates (n=42) born ≤34 weeks of gestational age were included in the study. Four feeding sessions—two in SLP and two in SEP— were analysed for each newborn. The level of saturation (SpO_2_) and heart rate, which are the parameters assessing the physiological stability, were measured in the studied newborns. The other factors that were examined to determine the quality of feeding included the total time of decline of SpO_2_ to ≤85%, level of the newborn’s alertness measured using the Neonatal Behavioral Assessment Scale, and the frequency of choking episodes. The proportion of milk consumed (volume of milk consumed relative to the volume expected) and the feeding duration as well as the total time of feeding session were recorded.

**Results:**

SLP was safer in terms of the frequency of choking episodes. Choking episodes were more frequently observed with feeding in SEP (*p*<0.001). Moreover, the proportion of milk consumed by infants was statistically significantly higher in SLP (*p*<0.046) compared to SEP. No significant differences in the other tested parameters were noted in infants fed in SLP and infants fed in SEP.

**Conclusions:**

This study demonstrated that SLP is effective in reducing the number of choking episodes during feeding. The proportion of milk consumed was better when the neonates were fed in SLP.

## Introduction

Improving the quality and safety of oral feeding is an important aspect of neonatal care. In premature babies, oral feeding and nourishment are considered a challenge as their neurological, respiratory, muscular and digestive systems are not fully mature and the possibilities of nutritional adaptation during extrauterine development are limited. Most preterm infants require support for oral feeding [1]. In particular, in premature babies born ≤34 weeks of gestational age, gastric tube feeding is more common and oral feeding is introduced gradually [2, 3].

The commencement of full oral feeding is often associated with adverse events, such as saturation decline, bradycardia or choking episodes, which are mostly related to the lack of proper coordination of sucking, breathing and swallowing [4]. All these events may cause stress in parents as well as newborns. Stress, in turn, affects the immature nervous system of newborns [5]. Discomfort experienced by parents while feeding a premature child can reduce their sense of competence and increase concerns about discharge and taking care of the infant on their own [6, 7]. Thus, it is important to identify solutions that can improve the quality of preterm infant feeding in hospital wards.

Optimal bottle-feeding position may contribute to improving oral feeding in immature infants. In neonatal care, bottle-feeding is followed either as a temporary solution until full oral feeding or mixed feeding is achieved [8], or when breastfeeding is impossible for some reasons [9]. However, bottle-feeding is an important part of premature baby care, as it usually includes the time when the baby starts taking food orally [9]. Due to immaturity, adverse events are more likely to occur during this period of feeding. This study aimed to assess the influence of two feeding positions—side-lying position (SLP) and semielevated position (SEP)—on the course of feeding, considering both qualitative and quantitative aspects of bottle-feeding.

## Material and methods

The study included a total of 42 neonates (n=42) who were hospitalised between August 2018 and April 2020 in neonatology ward. The neonates were born ≤34 weeks GA and had reached the age of at least 32 weeks of gestation on the day when the study began.

The inclusion criteria were cardiovascular cand respiratory stability and readiness for oral feeding as confirmed by a neurologopedic assessment. The included infants were transitioning from enteral nutrition to full oral feeding and fed orally at least 4–6 times within 24 hours. Parents provided informed consent for their infants to participate in the study.

The exclusion criteria included disorders that could significantly affect the feeding course, such as cleft lip and/or palate, facial paralysis, and/or congenital defects of the facial skeleton, diagnosed congenital abnormalities, metabolic diseases and a low Apgar score (<5 points at the fifth and tenth minute of the measurement). Furthermore, newborns were excluded if they were administered analgesics, anticonvulsants and sedatives, or if they were extubated <72 hours before the trial. Infants were also excluded if their parents refused to provide consent for participation in the study or did not prefer bottle-feeding for their child.

**Figure 1:**
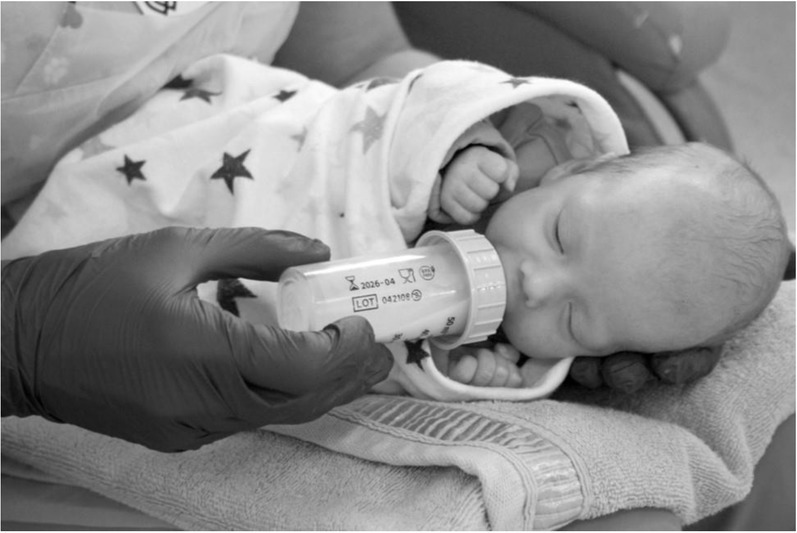
Infant fed in side-lying position.

**Figure 2:**
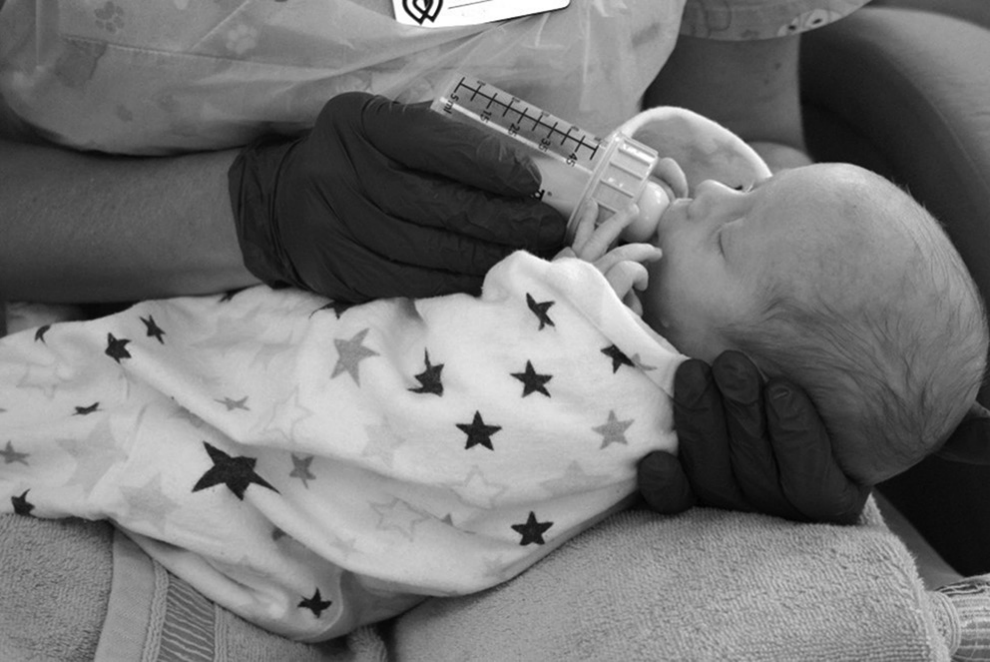
Infant fed in semielevated position.

The research was designed as a crossover, alternate study and randomised trial. Each infant was bottle-fed four times: two sessions in SEP and two sessions in SLP. The feeding position for the first session was randomly assigned and next the positions were alternated. The neonates positioned in SEP and SLP positions were placed on the researcher’s lap. In both positions, the child's lying angle was 30–45°. The main difference between the positions was that in the SEP a newborn was in the supine position, and in the SLP the baby was on the left side of its body. Figs 1 and 2 illustrate the feeding of infants in SLP and SEP. For one day only, two consecutive feeding sessions of an infant were included in the study in order to minimise fatigue.

The study included the analysis of the level of saturation (SpO_2_) and heart rate (HR; pulse oximeter data) and the level of newborn activity. These parameters were measured 2 minutes before feeding, at the third and tenth minute of feeding, at the end of feeding, and after 10 minutes of feeding. The proportion of milk consumed (volume of milk consumed relative to the volume expected) was measured at the tenth minute of feeding and at the end of feeding. The total time of decline of SpO_2_ to ≤85% and the feeding duration as well as the total time of feeding session were also recorded.

The occurrence of choking episodes also was noted. The choking episode was defined as discoordination of sucking, breathing, and swallowing during feeding manifested by coughing, interruption of feeding and the coexistence of additional factors characteristic of a choking episode, e.g. decrease in saturation, bradycardia, decrease in the activity of the newborn.

The level of activity was assessed in newborns using the six-point Neonatal Behavioral Assessment Scale, which is commonly known as Brazelton scale. The scores of this scale indicate the following: 1—quiet sleep, 2—active sleep, 3—drowsy, 4— quiet alert, 5—active alert, 6—crying [10].

The maximum time of feeding session was 40 minutes. The duration of feeding session was measured from the moment the newborn was picked up from bed, while the duration of feeding was measured from the moment of teat insertion into the baby’s mouth for the first time. Interruptions in feeding such as pauses for equalising the newborn’s breath or for burping were excluded from the measurement of total time of feeding, but the measurement of total time of feeding session included these events.

Throughout the study, the same type of bottle and teat were used. Each feeding session was recorded by a video camera.

## Statistical analysis

Statistical analysis was conducted using IBM SPSS v. 25 and Jamovi v1.2.8.The analyses performed in the study include frequency distribution analysis, basic descriptive statistics with Shapiro–Wilk’s test for normality of distributions, *t-*tests for independent samples, two-way independent mixed analyses of variance (ANOVAs) and chi-squared test of independence.

## Results

The age of infants at birth was 28–34 weeks GA [mean=31.34; standard deviation (SD)=1.89], and age at the time of beginning of the study was 32+6/7–37+6/7 weeks (mean=34.05; SD=3.32). The birth weight of infants was 1100–2500 g (mean=1690; SD=360).

### Oxygen saturation

There was no significant difference between the two study groups (*p*=0.128). Considering the SEP the highest mean value was noted two minutes before the feeding in SEP (96.89%; SD=2.5%), while the lowest value was recorded at the end of feeding (94.19%; SD=3.66%). For the SLP, the highest mean value was observed two minutes before feeding (97.83%; SD=1.74%), while the lowest one was detected in the tenth minute of feeding (95.31%; SD=3.67%).

### Heart rate

There was no significant difference between the two study groups (*p*=0.639). For the SEP, the highest mean value was observed at the end of feeding (169.85/bpm; SD=9.94/bpm), while the lowest one was notice 10 minutes after it finished (159.90/bpm; SD=9.26/bpm). In the SLP, the largest mean value was observed in tenth minute of feeding (166.67/bpm; SD=9.77/bpm), while the lowest one was detected 10 minutes after feeding (159.25/bpm; SD=12.85/bpm).

### Proportion of food intake

**Figure 3:**
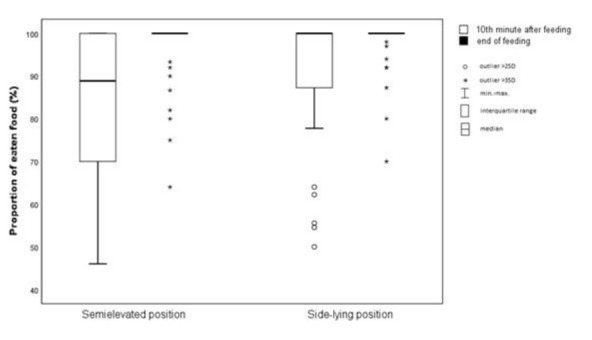
Measurements of the proportion of eaten food.

There was significant difference between the two study groups (*p*=0.046). The infants consumed a statistically significantly more volume of food in the second measurement point (mean=97.30%; SD=6.99%) compared to the first (mean=87.42%; SD=15.97%). Regarding the child’s position during feeding, a statistically significant difference was observed between SEP (mean=90.26%; SD=10.67%) and SLP (mean=94.46%; SD=8.16%). The compared means are presented in Figure 3.

**Figure 4:**
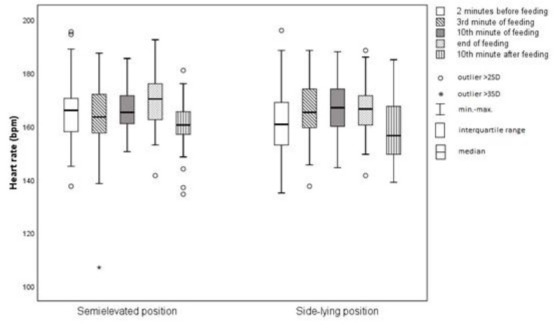
Average heart rate.

**Figure 5:**
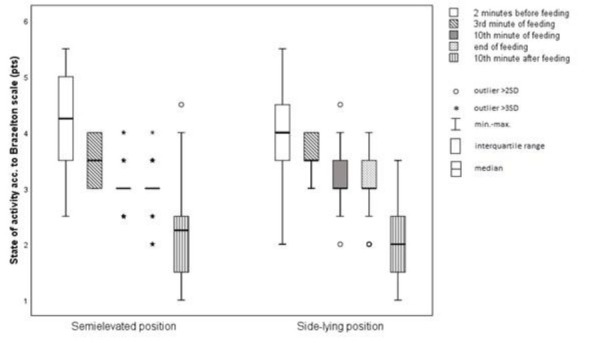
Average activity level of infants.

**Figure 6:**
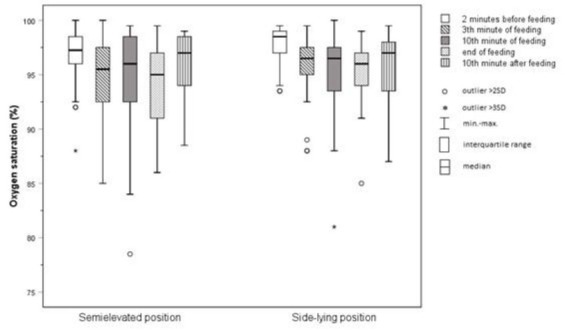
Mean levels of blood saturation.

### Choking episodes

There was statistically significant difference between the study groups in the number of choking episodes (*p*=0.001). Total numbers of choking episodes which occurred during study in SEP was 30 times, and 13 times in SLP. The compared means are presented in Table 1.

**Table 1:** Cross table for infant position during feeding and choking episodes

Choking episodes		Position	
		Semielevated	Side-lying
0	*n*	12	29
	%	29,3%	70,7%
		(-3,7)	(3,7)
1	*n*	18	13
	%	58,1%	41,9%
		(1,1)	(-1,1)
2	*n*	12	0
	%	100,0%	0,0%
		(3,7)	(-3,7)

Note. Standarised adjusted residuals were shown in parenthesis χ^2^ (2) = 19,85; *p* < 0,001; *V_c_* = 0,49

### Other factors

The average time periods of feeding, feeding sessions and declines of saturation to ≤85% were compared between the infants fed in SEP and infants fed in SLP. No statistically significant differences were found between the analysed groups in time periods of feeding (*p*=0.562), feeding sessions (*p*=0.372) and declines of saturation to ≤85% (*p*=0.052). Moreover, there was no statistically significant difference between the two study groups in the level of activity (*p*=0.885).

**Table 2:** Comparative table of feeding times and feeding sessions between the semielevated and side-lying positions

	*t*	*df*	*p*
Duration of feeding	0,58	82	0,562
Duration of feeding session	0,90	82	0,372

*t – t*-test result; *df –* degrees of freedom; *p* – significance

**Table 3:** Comparison of the periods of saturation declines to ≤85% level between the semielevated and side-lying positions

	*t*	*df*	*p*
Saturation declines to ≤85% level	1,97	82	0,052

*t – t*-test result; *df –* degrees of freedom; *p* – significance

## Discussion

In the last 20 years, several studies were performed with an aim of finding solutions to improving the quality of breastfeeding [11] and bottle-feeding [12]. An important factor analysed in studies was the relationship between the position during feeding and the course of feeding [13, 14, 15, 16]. The present study assessed the influence of position on the course of feeding. In addition to the standard factors, it also analysed the incidence of choking episodes during feeding.

In preterm infants, choking episodes are fairly common during the transition from nasogastric tube feeding to full oral feeding. Although choking does not put the health and life of a preterm newborn at risk, it can cause desaturation, apnea, or bradycardia. Severe choking may lead to food aspiration to the respiratory tract [17] and increase the risk of aspiration pneumonia [18]. Choking episodes lead to stress in a child, which in turn affects brain development and the immature immune and neuroendocrine system [5]. The occurrence of choking episodes is also stressful for parents. Stress in mothers may affect their lactation and interaction with the child [1920]. Stressful feeding has a negative impact on the sense of competence of parents in the care of the newborn [21]. The results of the present study revealed that newborns fed in SLP experienced fewer choking episodes compared to newborns fed in SEP. SLP can also reduce the adverse effects associated with choking episodes, including both direct effects, such as apnea and desaturation, and indirect effects, such as stress, in preterm newborns and their parents.

Choking episodes may be associated with unfavourable oral feeding experience. Studies indicate that premature babies are more likely to develop feeding disorders in early childhood [22, 23], which may be linked to an inappropriate approach to learning to suck [24, 25]. Thus, feeding preterm infants in SLP may reduce their risk of feeding disorders in the later stages during the development of eating behaviours. This can be justified by a lower incidence of adverse events during feeding.

In previous studies assessing the benefits of the lateral position, the newborns were fed in slightly different variants of SEP and SLP [13, 14, 15, 16]. The differences associated with these positions can be significant for the feeding process. The study by Park et al. [16] showed the greatest differences in feeding-related parameters between SEP and SLP, in which the lateral position was defined as a semielevated SLP. The results of their study indicated that infants fed in semielevated SLP were slightly more stable compared to infants fed in semielevated supine position. The semielevated supine position defined by Park et al. was also significantly different from the SEP defined in the present study, as in their study the newborn was placed parallel to the knees of the person nursing the baby [16]. Similarly, the feeding positions assessed in the studies of Dawson et al. [14] (cradle-hold position and SLP) and Girgin et al. [15] (semielevated SLP and semielevated supine position) were different from the positions assessed in the present study.

Clark et al. analysed the most similar feeding position to SLP in their study [13]. However, the elevated SLP used in the cited study was not the same as the SLP used in this study. The differences also applied to the semiupright position, as in the study by Clark et al. whereby the newborn was supported mainly around the head and the top of the shoulder girdle and the head was also higher, almost at an angle of 90° [13]. The differences in the SEP and SLP assessed in studies carried out so far, and the observed influence of these positions in the course of bottle-feeding, are so significant that each of the studies, in a sense, concerned different feeding positions.

Interesting conclusions about the influence of position on bottle-feeding of newborns can be drawn by analysing the results of the study by Girgin et al. [15] and comparing those results with the results of the present research. The study by Girgin et al. indicated that newborns fed in semielevated SLP (n=38) demonstrated better physiological stability compared to infants fed in the semielevated supine position (n=42). In semielevated SLP, children had higher SpO_2_ and lower HR [15]. This suggests that the physiological stability parameters of newborns can be improved by positioning them at a slightly greater angle to the ground than that followed in this study (45–60° vs 30–45°). However, this requires further research, and other factors should also be analysed in detail. According to Girgin et al., positioning at higher angles relieves the respiratory and cardiovascular system of the newborn. However, in premature babies with digestive disorders, higher position may exert stress on the digestive system. Nonetheless, the study by Girgin et al. indicates that the improvement of physiological stability is influenced not only by the child’s position but also by the angle of the position relative to the ground [15].

At this point, it is important to emphasise that parents should be able to independently feed their child before the child is discharged from the hospital [26]. Anxiety and incompetence of parents may result in a reluctance to independently take care of the newborn and delayed discharge from hospital [6, 27]. This may contribute to adverse consequences resulting from prolonged hospitalisation, such as an increased risk of nosocomial infections and unnecessary exposure to diagnostic testing, including blood sampling, as well as higher hospitalisation costs.

The use of a feeding position that allows preterm infants to consume larger portions of food without extending the feeding duration could reduce the need for nasogastric tube feeding. Such a position could thus reduce the risk of adverse effects associated with tube feeding, including desaturation, irritation of the esophageal and gastric mucosa, and rare injuries such as esophageal perforation and iatrogenic complications [28]. The present study performed an analysis of the volume of food consumed by infants. The difference in consumed food volume observed in the tenth minute was statistically insignificant, but the difference observed at the end of the feeding was statistically significant (*p*<0.046). It can, therefore, be assumed that fatigue causes newborns to consume smaller amounts of food when fed in SEP. Thus, SLP can be considered to be associated with decreased fatigue. When attempting to justify the impact of SLP on fatigue reduction, it can be pointed out that the support plane is larger in SLP compared to SEP, which may result in greater muscle relaxation within the newborn’s body and less pressure of peritoneal cavity content on the diaphragm and subsequently on the pleural cavity. This, in turn, could lead to a reduction in the indirect negative influence on the functioning of the respiratory system. There is no scientific evidence available to support this hypothesis. However, it does appear to be a valid explanation for reduced fatigue in SLP, which enables preterm infants to consume larger amounts of food. In addition, less pressure on the peritoneal cavity in SLP may be associated with lower pain and discomfort caused by digestive problems in preterm newborns. Moreover, Dawson et al. observed that infants fed in SLP tended to consume more food in comparison to the cradle-hold position. The authors compared the feeding performance of newborns (n=25) of similar maturity (<34 weeks GA) in both SLP and cradle-hold positions and observed that a greater amount of food was consumed in SLP [14].

It is also worth mentioning that SLP is similar to the position used for breastfeeding. Placing preterm infants in SLP during bottle-feeding may help them to adapt to feeding in the commonly used breastfeeding positions. The mothers of infants enrolled to this study were encouraged to breastfeed and informed about the pro-health effects of breastfeeding for mother and child [29].

### Key points

1. The study demonstrated that SLP is effective in reducing the number of choking episodes during feeding.
2. The neonates fed in SLP consumed a higher proportion of milk compared to neonates fed in SEP.
3. Based on the benefits of bottle-feeding in SLP indicated by this study, it can be suggested that this position should be more frequently used for feeding preterm infants.
